# Abatacept enhances blood regulatory B cells of rheumatoid arthritis patients to a level that associates with disease remittance

**DOI:** 10.1038/s41598-021-83615-0

**Published:** 2021-03-11

**Authors:** Maha Fahad Alenazy, Fatemeh Saheb Sharif-Askari, Mohammed A. Omair, Mohammad S. El-Wetidy, Maha A. Omair, Hussam Mitwalli, Saleh Al-Muhsen, Abeer Al-Masri, Qutayba Hamid, Rabih Halwani

**Affiliations:** 1 Immunology Research Lab, College of Medicine, King Saud University, Riyadh, Saudi Arabia; 2 Department of Physiology, College of Medicine, King Saud University, Riyadh, Saudi Arabia; 3 Sharjah Institute of Medical Research, University of Sharjah, Sharjah, United Arab Emirates; 4 Rheumatology Unit, Department of Medicine, College of Medicine, King Saud University, Riyadh, Saudi Arabia; 5 Department of Statistics and Operations Research, College of Science, King Saud University, Riyadh, Saudi Arabia; 6 Department of Pediatrics, College of Medicine, King Saud University, Riyadh, Saudi Arabia; 7 Department of Clinical Sciences, College of Medicine, University of Sharjah, Sharjah, United Arab Emirates; 8 Meakins-Christie Laboratories, Research Institute of the McGill University Health Center, Montreal, QC Canada; 9 Prince Abdullah Ben Khaled Celiac Disease Chair, College of Medicine, King Saud University, Riyadh, Saudi Arabia

**Keywords:** Immunology, Translational immunology

## Abstract

Abatacept, an inhibitor of CD28 mediated T-cell activation, has been shown to be effective in controlling inflammation during rheumatoid arthritis (RA). However, its effects on immune regulatory B and T cells (Bregs and Tregs) has not been fully explored. Thirty-one RA patients treated with abatacept for ≥ 6 months along with 31 RA patients treated with other modalities as well as 30 healthy controls were recruited. Of these 62 RA patient, 49 (79%) were females with a mean age of 54 ± 12 years and disease duration of 10 ± 6 years. The blood levels of Tregs and Bregs and their production of immunosuppressive cytokines, were determined using FACS analysis and Luminex Multiplex assay. Treatment with abatacept significantly enhanced the blood level of IL-35^+^ IL-10^+^ Bregs (P = 0.0007). Their levels were higher in the blood of remitted patients (DAS28-CRP < 2.6) compared to the unremitted ones (P = 0.0173), 6 months following abatacept treatment initiation. Moreover, abatacept treatment significantly enhanced the blood levels of LAG3^+^ conventional and unconventional Tregs of RA patients. This increase in the blood levels of Bregs and Tregs was accompanied with an elevated serum level of IL-35 and IFN-β in abatacept-treated patients. Therefore, Abatacept efficiency to achieve remittance in RA could be attributed, in part, to its ability to enhance immune regulatory cells, especially IL-35^+^ IL-10^+^ Bregs.

## Introduction

Abatacept is a chimeric recombinant protein composed of the extracellular domain of human cytotoxic T-lymphocyte antigen 4 (CTLA4) fused to the Fc region of the immunoglobulin IgG1^1^. It was shown to be effective for the treatment of moderate to severe rheumatoid arthritis (RA) patients with an inadequate response to conventional synthetics disease modifying anti-rheumatic drugs (csDMARDs), including methotrexate or a TNF-α therapy^2^. It competes with CD28 for CD80 or CD86 binding; and thereby, selectively ameliorate T-cell activation^3^. Besides its CD28 dependent T cell inhibitory mechanism, several reports have shown that abatacept enhances proliferation and suppressive function of regulatory T (Tregs) cells^4–6^. Vogel et al. showed that CTLA‐4Ig therapy resulted in an initial rise and activation of Tregs in spite of CD28 costimulatory blockage^5^. Moreover, Alvarez-Quiroga et al.^6^ detected an enhanced suppressive function of Tregs in the blood of RA after abatacept therapy. These Tregs predominantly produce a number of immune suppressors such as IL-10 and IL-35 cytokine, that play key roles in the control of chronic inflammation during autoimmune diseases^7^.

On the other hand, regulatory B cells (Bregs) have been shown to play a key role in controlling inflammation during chronic inflammatory diseases^8^. IL-10 producing Bregs inhibit the differentiation of T helper type 1 (Th1) and T helper type 17 cells (Th17) and decrease proinflammatory cytokines production by dendritic cells^9^. Similarly, IL-35^+^ producing Bregs were shown to be potent suppressors of inflammation during experimental autoimmune uveitis^8,10^.

The frequency of IL-10^+^ Bregs were shown to be reduced in RA patients^11,12^. However, the role of IL-35-producing Bregs in RA is not clearly elucidated. Although the effect of several biologics used for the treatment of RA on regulatory cells has been investigated^13–15^, how abatacept treatment may regulate the levels of Bregs, conventional Tregs (cTregs) and unconventional Tregs (uTregs) is not largely known^16,17^. Thus, besides its direct role in suppressing T cells activation, it is crucial to assess the drug’s ability to induce regulatory B and T cells as a CD28 independent mechanism of controlling chronic inflammation during RA. In this study, we determined the effect of abatacept treatment on the levels of peripheral blood regulatory cells (Bregs and Tregs) in RA patients; compared to treatment with csDMARDs or other biologic DMARDs (bDMARDs). Our finding unraveled an indirect suppressive effects of abatacept and may rationalize its use for other chronic inflammatory diseases.

## Materials and methods

### Patients and control subjects

Adult RA patients fulfilling the American College of Rheumatology (ACR)/European League Against Rheumatism (EULAR) 2010 classification criteria with a disease duration more than 1 year were recruited; along with age and sex matched healthy controls (Mean Standard Deviation [SD] age of 54 (12) years, 79% female for RA; and mean (SD) age of 48 (5) years, 73% female for the healthy controls) from January 2019 to March 2019. Patients with end stage liver diseases, end stage renal disease, prior diagnosis of malignancy, active infection, overlap with lupus or systemic sclerosis were excluded. Patients’ demographics, disease characteristics and current medications were collected from records at the time of recruitment. Disease severity of each patient was evaluated based on the 28-joint disease activity score-C-reactive protein (DAS28-CRP)^18,19^. Patients were divided into two groups: abatacept-treated group and RA control group treated with either csDMARDs or bDMARDs. Patients recruited in the abatacept group should have received the drug for ≥ 6 months. The duration of treatment with abatacept was 13 months on average (95% CI 8–17 months). Response to abatacept was evaluated using the EULAR response/disease state criteria^20^. The Ethics Committee of King Khalid University Hospital, King Saud University, Riyadh, Saudi Arabia approved this study (IRB No. E-16-2102). Written informed consent was obtained from all study participants prior to inclusion. The design and procedures of the study were conducted in accordance with the Declaration of Helsinki.

### Cell preparation and flow cytometry analysis

Peripheral blood mononuclear cells (PBMCs) were isolated from blood samples using a Ficoll gradient (Axis Shield, Norway). Unstimulated PBMCs were stained with APC anti-human CD19 antibody (BioLegend cat #302212), APC/Cyanine7 anti-human CD138 antibody (BioLegend cat #356528), PerCP/Cyanine5.5 anti-human CD1d antibody (BioLegend cat #350312), APC anti-human CD25 antibody (BioLegend cat #302610), and PE anti-human CD223 (LAG-3) antibody (BioLegend cat #369306). After surface staining, cells were stained with PE anti-human EBi3 antibody (BioLegend cat #360904) and PE/Cy7 anti-human IL-10 antibody (BioLegend cat #501420), FITC anti-human FOXP3 antibody (BioLegend cat #320106). Corresponding isotype controls were used for each antibody. All cells were analyzed with BD LSR II flow cytometer using DIVA software version 8.0.

### Luminex multiplex assay of serum IL-35 and IFN-β

Serum IL-35, IL-10, IFN-β, IL-17, IL-1β, and HMGB1 cytokines concentrations in 62 RA patients were determined using commercially available human ELISA kit (MILLIPLEX MAP Human Cytokine/Chemokine Magnetic Bead Panel IV, cat # HCYP4MAG-64K-0). Assays were preformed strictly following the manufacturer’s instructions. All samples were measured in duplicates.

### Statistical analysis

Statistical comparisons were performed by using t-test if the data was normally distributed, or Mann–Whitney U-tests if the data was skewed. The analysis was performed using SPSS Version 26 (IBM Corporation, Chicago, USA) and Graphpad Prism 8 (GraphPad Software Inc., San Diego, USA). A two-sided P value ≤ 0.05 was considered statistically significant.

## Results

### Demographics and disease activity

A total of 62 patients (31 in each group) and 30 controls were recruited. Mean (SD) age 54 (12) and disease duration were 10 (6) years, respectively. Seropositivity was detected in 39 (63%) and mean (SD) DAS28-CRP was 2.45 (1) with no significant difference between the 2 subgroups. Abatacept treatment achieved clinical remission defined as DAS28-CRP < 2.6 in 71% of the patients after 6 months of therapy. Demographic and clinical features of the RA patients are detailed in Table 1.

**Table 1:** Demographic and clinical characteristics of abatacept-treated and untreated RA patients.

Characteristics	All RA (n = 62)	RA not on abatacept (n = 31)	Abatacept-treated (n = 31)	P-value
**Demographic characteristics**				
Female	49/62 (79%)	27/31 (87%)	22/31 (74%)	NS
Age, mean (SD), y	54 (12)	55 (13)	53 (12)	NS
**Comorbidities**				
Asthma	17/62 (27%)	11/31 (35%)	6/31(19%)	NS
Osteoporosis	11/62 (18%)	5/31 (16%)	6/31 (19%)	NS
Sjögren Syndrome	4/62 (6%)	3/31 (10%)	1/31 (3%)	NS
Type 2 Diabetes Mellitus	14/62 (23%)	7/31 (23%)	7/31 (23%)	NS
CVD	14/62 (23%)	9/31 (29%)	5/31 (16%)	NS
**Clinical measures**				NS
Disease duration, mean (SD), y	10 (6)	11 (7)	9 (6)	NS
RF positive, No. (%)	39/62 (63%)	21/31 (72%)	18/31 (69%)	NS
Anti-CCP positive, No. (%)	22/62 (35%)	10/31 (32%)	12/31 (39%)	NS
Disease Activity Score (DAS28), mean, SD	2.45 ± 1	2.56 ± 1	2.34 ± 1	NS
^a^DAS 28 < 2.6	36/62 (58%)	14/31 (45%)	22/31 (71%)	0.039
Tender joint count (0–28), mean, SD	1.85 (4)	1.96 (3)	1.74 (5)	NS
Swollen joint count (0–28), mean, SD	1.59 (2)	1.83 (2)	1.35 (2)	NS
Patient’s assessment of pain, visual analogue scale (0–10)	2.79 (3)	3.38 (3)	2.19 (3)	NS
**Medications**				
Prednisolone	16/62 (26%)	5/31 (16%)	11/31 (35%)	NS
^b^csDMARDs	29/62 (47%)	14/31 (39%)	15/31 (48%)	NS
^c^bDMARDs	15/62 (24%)	15/31 (48%)	–	–

*Anti-CCP* Anti-cyclic citrullinated peptide, *csDMARDs* conventional synthetic disease modifying antirheumatic drugs, *bDMARDs* biologic disease modifying antirheumatic drugs, *RA* Rheumatoid arthritis, *RF* Rheumatoid factor, *SD* Standard Deviation, *NS* not significant. Statistics significant: *P* < 0.05.

^a^DAS28 score lower than 2.6 indicates remission.

^b^csDMARDs involved methotrexate or leflunomide.

^c^bDMARDs involved rituximab (n = 6), etanercept (n = 1), adalimumab (n = 1), tocilizumab (n = 6), or tofacitinib (n = 1).

### Abatacept enhanced the level of LAG3^+^ Tregs in peripheral blood of RA patients

**Figure 1:**
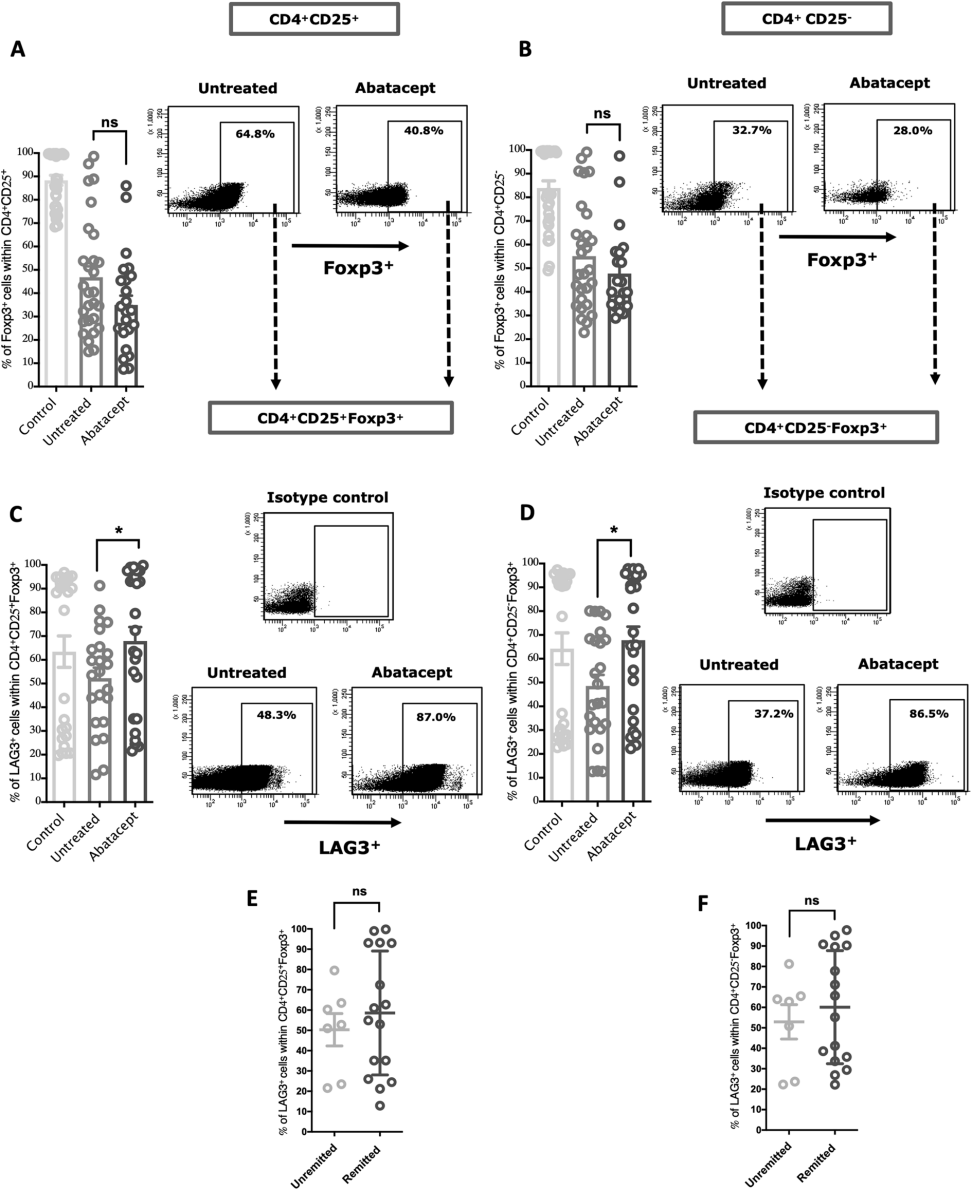
Effects of the abatacept on the frequency of conventional (cTreg) and unconventional Tregs (uTreg) in the PBMCs of RA patients. (**A**,**B**) Percentage of cTreg (Foxp3^+^ cells within CD4^+^CD25^+^) and uTreg (Foxp3^+^ cells within CD4^+^CD25^−^) in the PBMCs of abatacept-treated and untreated RA patients, and in the healthy controls. Representative FACS data showing no significant difference in the frequency of these cells in PBMCs following treatment with the abatacept. (**C**,**D**) Percentage of LAG3^+^ cTreg (LAG3^+^ cells within CD4^+^CD25^+^Foxp3^+^) and LAG3^+^ uTreg (LAG3^+^ cells within CD4+CD25^−^Foxp3^+^) cells in the PBMCs of abatacept-treated and untreated RA patients. Representative FACS data showing a significant increase in the population of these Tregs in PBMCs following treatment with the abatacept. (**E**,**F**) Percentage of LAG3^+^ cTregs and LAG3^+^ uTregs in the PBMCs of remitted and unremitted abatacept-treated RA patients. Representative FACS data shows an increasing pattern in the percentage of these Tregs in PBMCs following treatment with the abatacept. DAS28-CRP score lower than 2.6 indicates remission. Events were recorded and analysed by using BD FACSDiva version 8.0. Two-way comparison was done using t-test. *ns* non-significant. *P < 0.05.

A variety of Treg subtypes have been shown to be involved in controlling autoimmune inflammation, including the conventional CD4^+^CD25^+^Foxp3^+^ Tregs (cTreg) as well as the unconventional CD4^+^CD25^-^Foxp3^+^ Tregs (uTreg)^21^. Lymphocyte activation gene 3 (LAG3) expression on these Tregs was associated with interleukin-10-production^22^. LAG3^+^ Tregs has been reported to attenuate joint inflammation in active arthritis^23^. We hence evaluated, in RA patients, the effect of abatacept treatment on the blood levels of cTregs and uTreg expressing, or not, LAG3. No increase was observed in the blood levels of cTregs and uTregs cell population following treatment (Fig. 1A; P = 0.063, and Fig. 1B; P = 0.231, for cTreg and uTreg, respectively; Supplementary Fig. 1). However, treatment with abatacept increased the blood levels of LAG3^+^ cTreg (LAG3^+^ cells within CD4^+^CD25^+^Foxp3^+^) and LAG3^+^ uTreg (LAG3^+^ cells within CD4^+^CD25^-^Foxp3^+^) in treated compared to untreated RA patients (Fig. 1C; P = 0.0414, and Fig. 1D; P = 0.0122, for LAG3^+^ cTreg and LAG3^+^ uTreg, respectively). These results indicated that beside reducing T cell activation, abatacept expanded LAG3^+^ Tregs, the potent subtype of Tregs. We have also noticed an increasing trend in levels of these Tregs in the blood of remitted patients, defined as DAS28-CRP < 2.6, although not to a significant level (Fig. 1E; P = 0.525, and Fig. 1F; P = 0.553, for LAG3^+^ cTreg and LAG3^+^ uTreg, respectively).

### Abatacept increases peripheral blood level of IL35^+^IL-10^+^ Bregs in RA patients

Abatacept is known to downregulate the level of B cells^24^. We first examined the level of these cells in the treated and untreated patients. As expected, the peripheral blood level of CD19^+^ cells were elevated in RA patients not on abatacept compared to the healthy controls (Supplementary Fig. 2B; P = 0.0044); while treatment with abatacept dramatically reduced their levels (Supplementary Fig. 2B; P = 0.0169).

We next investigated how abatacept may regulate the level and function of the immune suppressive subtype of B cells, Bregs. The hallmark of Breg-mediated suppression is the production of anti-inflammatory cytokine, including IL-10, transforming growth factor-β (TGF-β), and IL-35. Breg cells express different surface markers, such as CD1d, CD21 (EBi3), and CD138. CD1d is a marker that is expressed by the majority of Breg subsets inducing those producing IL-10, and to a lesser extent IL-35^25^. EBi3^+^ is a subunit of IL-35 cytokine and have been used as a marker for IL-35 production^26^.

Low CD1d levels were found to be associated with higher disease-severity defined as DAS28 score^27^. We, hence, evaluated the blood levels of CD19^+^ CD1d^+^ in abatacept-treated and untreated RA patients, and in the healthy controls. As expected, the blood level of CD19^+^CD1d^+^ of RA patients was significantly lower compared to those of healthy controls (P = 0.0001); notably, their blood levels increased significantly after treatment with abatacept (Supplementary Fig. 3A; P = 0.0021). However, no association was observed of this Bregs population with the level of remittance in RA patients (DAS28-CRP < 2.6) (Supplementary Fig. 3B; P = 0.6791).

**Figure 2:**
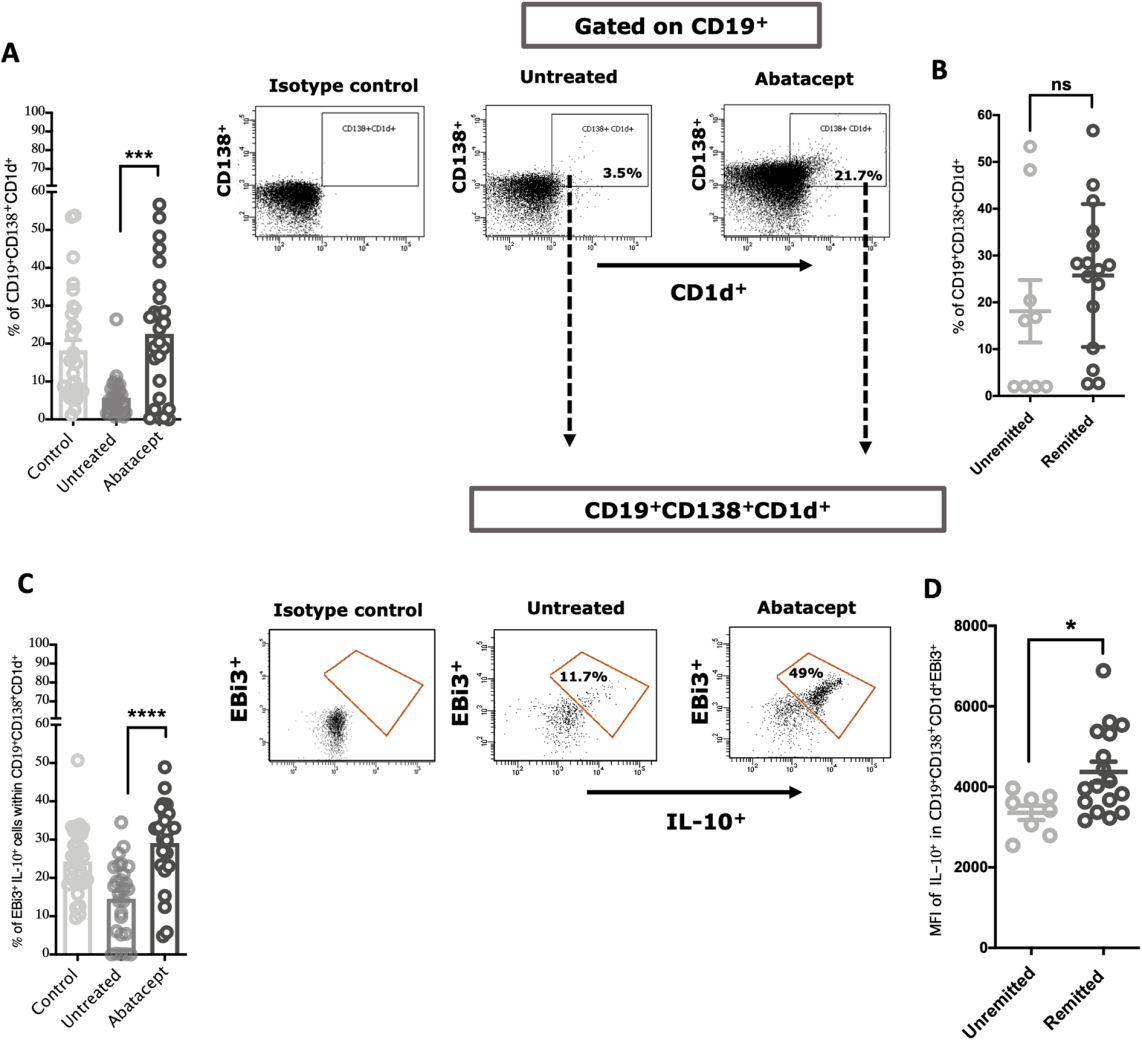
Effects of the abatacept on the frequency of regulatory B cells in the PBMCs of RA patients. (**A**) Percentage of CD138^+^CD1d^+^ Bregs in the PBMCs of abatacept-treated and untreated RA patients. Representative FACS data showing an increase in the percentage of CD138^+^CD1d^+^ Bregs in PBMCs following treatment with the abatacept. (**B**) There is an increasing trend in the percentage of this Breg subtype in remitted compare to unremitted abatacept-treated groups. (**C**) Percentage of IL-35^+^IL-10^+^ Bregs (EBi3^+^IL-10^+^ cells within CD19^+^CD138^+^CD1d^+^) in the PBMCs of abatacept-treated and untreated RA patients. Representative FACS data showing an increase in the percentage of IL-35^+^IL-10^+^ Bregs in PBMCs following treatment with the abatacept. (**D**) The level (MFI) of IL-10^+^ in CD138^+^CD1d^+^EiB3^+^IL-10^+^ Bregs was significantly increased in remitted compared to unremitted abatacept-treated groups. DAS28-CRP score lower than 2.6 indicates remission. Events were recorded and analysed by using BD FACSDiva version 8.0. Two-way comparison was done using t-test. *ns* non-significant. *P < 0.05, **P < 0.01, ***P < 0.001, ****P < 0.0001.

We then evaluated the blood level of CD19^+^CD138^+^CD1d^+^ Bregs in abatacept-treated and untreated RA patients. Interestingly, abatacept significantly expanded the level of this cell population in the blood of treated RA patients [Fig. 2A; P = 0.0001]. The level of CD19^+^CD138^+^CD1d^+^ Bregs was higher in blood of remitted RA patients (DAS28-CRP < 2.6), however, not to a significant level (Fig. 2B; P = 0.6791). It is noteworthy that the frequency of plasmablast (CD19^+^CD138^+^) was not significantly changed following treatment with abatacept (Supplementary Fig. 4) indicating that the effect of abatacept is specific to regulatory cells.

We next examined the effect of abatacept on the CD138^+^CD1d^+^ Bregs producing anti-inflammatory cytokines, as these are the most potent subtypes of Bregs. Despite progress made in understanding the importance of Breg-mediated IL-10 subsets in various autoimmune disorders^25^, our understanding of the effect of bDMARDs on the frequency and function of these Bregs in RA is limited. It was not until recently that abatacept was shown to induce CD24^hi^ CD27^+^ memory B cells (also producing IL-10)^28,29^. The effect of abatacept treatment on IL-35 and/or IL-10 producing Breg subtypes^25^, including CD138^+^ CD1d^+^ was not investigated, especially in the context of RA. Here, treatment with abatacept significantly enhanced the level of IL-35^+^IL-10^+^ Bregs (EBi3^+^IL-10^+^ cells within CD19^+^CD138^+^CD1d^+^) in peripheral blood of treated compared to RA patients not on abatacept (Fig. 2C; P = 0.0007). Interestingly, the blood level of IL-35^+^IL-10^+^ Bregs in remitted patients (DAS28-CRP < 2.6) was significantly higher compared to that of unremitted (Fig. 2D; P = 0.0173).

Furthermore, compared to patients treated with other bDMARDs, including tocilizumab (n = 6), a humanized anti-IL6 receptor antibody, and rituximab (n = 6), an anti-CD20 antibody for a similar duration (> 6 months), abatacept significantly enhanced IL-35^+^IL-10^+^ Bregs blood levels (Supplementary Fig. 5A; P = 0.0055). Moreover, the blood levels of LAG3^+^ cTregs or LAG3^+^ uTregs were higher following abatacept treatment when compared to other bDMARDs, although not to a significant level (Supplementary Fig. 5B and C).

On the other hand, the recruited patients were on abatacept for an average of 13 months. To answer if the duration of therapy would affect the frequency of investigated regulatory immune cells, we compared the average percentage of IL-35^+^IL-10^+^ Bregs, LAG3^+^ cTregs and LAG3^+^ uTregs in patients who were on therapy for more or less than 13 months. As presented in Supplementary Fig. 5D–F, there was no significant difference in the average frequency of IL-35^+^IL-10^+^ Bregs, LAG3^+^ cTregs and LAG3^+^ uTregs between these two groups.

### Elevated serum IL-35 and IFN-β levels in abatacept-treated patients

**Figure 3:**
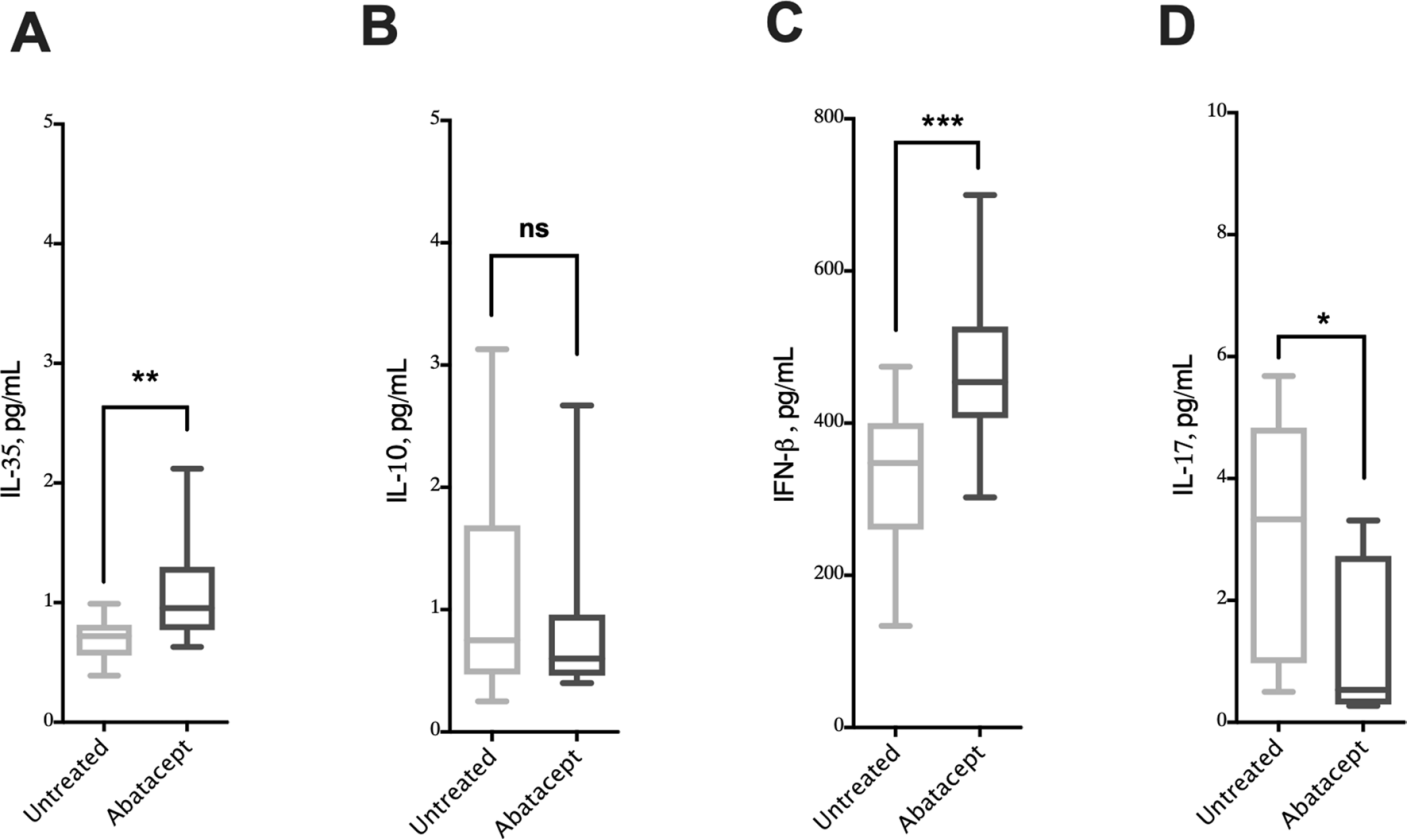
Effects of the abatacept on the cytokine levels in the serum of RA patients. (**A**–**D**) Serum levels of IL-35, IL-10, IFN-β and IL-17 in abatacept treated and untreated groups. Data show an increase in IL-35 level, no significant change in IL-10 level, increase in IFN-β levels, and a decrease in IL-17 level in the serum of abatacept treated compared to untreated RA groups. The cytokine levels were estimated using a human Luminex assay. Two-way comparison was done using t-test. *ns* non-significant. *P < 0.05, **P < 0.01, ***P < 0.001, ****P < 0.0001.

IL-10 and IL-35 cytokines have been reported to regulate arthritis pathogenesis and attenuate inflammatory joint symptoms^30,31^. We, hence, evaluated the serum levels of these cytokines in the serum of abatacept-treated and untreated RA patients using ELISA assay. Notably, treating with abatacept significantly increased the serum level of IL-35 (Fig. 3A; P = 0.0013), but not IL-10 (Fig. 3B; P = 0.8922).

IFN-β has been reported to be a gold standard treatment in some autoimmune disorders including, multiple sclerosis^32^. IFN-β- immune mediated mechanism is believed to be through modulation of B cell function^33^, expanding Foxp3^+^Tregs^34^, and enhancing CTLA-4 expression^35^. Abatacept reduces IFN-γ release in RA^36^, but its regulation of IFN-β production has not been investigated. Interestingly abatacept-treated patients had significantly elevated serum levels of IFN-β compared to RA patients not on abatacept [Fig. 3C; P = 0.0008]. The increased levels of IFN-β could hence contribute to the control of RA pathogenesis. Of note, treatment with abatacept suppressed blood levels of IL-17 [Fig. 3D; P = 0.0013]. However, no change in the serum levels of other RA-related pro-inflammatory cytokines such as IL-1β and HMGB1, were observed (Supplementary Fig. 6).

Interestingly, IL-10 serum levels of patients treated with abatacept correlated positively with IL-35^+^IL-10^+^ Bregs and LAG3^+^ cTregs, while serum levels of IL-35 and IFN-β of these patients correlated with the blood levels of conventional and unconventional Tregs expressing LAG3^+^ (Supplementary Table 1).

## Discussion

Regulatory T cells (Tregs) are functionally defective in patients with RA and believed to be at the center of RA pathogenesis^37^; therefore, restoring Treg levels in RA may control inflammation and restore tolerance in these patients. In an *invitro* study, abatacept was shown to enhance LAG3 expression on naïve CD4^+^ T cells, which led to increase CD4^+^ T cells suppressive activity^38^. In this study we have observed a significant elevation in the blood levels of LAG3 + cTreg or/and uTreg of RA patients 6 months following treatment with abatacept.

In addition, treatment with abatacept significantly enhanced the blood level of IL-35^+^ IL-10^+^ Breg in remitted RA patients. To our knowledge, this is the first study to report that in blood of RA patient, importantly, measured 6 months following treatment initiation. This suggests that this potent IL-35 and IL-10 producing Breg subtype may contribute to the therapeutic effect of abatacept and, more interestingly, to its ability to control disease severity and achieve remittance in RA patients. These results are in line with an invitro study showing abatacept ability to expand IL-10^+^ producing Bregs^17^. In regards to its effect on other Breg subpopulations, a high frequency of CD24^hi^ CD27^+^ memory B cells (also producing IL-10) was associated with DAS28 remittance in RA patients at 6 months treatment with abatacept compared to other anti-cytokine therapy such as anti-TNF drugs^28^. Moreover, abatacept treatment have been shown to significantly reduce CD19^+^ B cell levels in the blood of remitted RA (DAS28-CRP < 2.6), but not in unremitted patients (Supplementary Fig. 2). Other studies also report an increase in Treg frequency over the course of anti-TNF^39,40^ and abatacept^38,40^ treatment, but none has linked the levels to DAS28 remittance. Here, we have observed an increase in LAG3^+^ cTreg and uTreg blood levels of remitted RA following 6 months treatment with abatacept (Fig. 1E,F).

The mechanism by which abatacept enhances Treg and Breg differentiation and activation, however, is not fully understood. Binding of abatacept to B7 is believed to trigger dendritic cells to express indoleamine 2,3 dioxygenase, a tryptophan-catabolizing enzyme that induces and activates CD4^+^ CD25^+^ Treg^41,42^. Moreover, treatment of CD4^+^ T cells isolated from RA patients with abatacept enhanced their expression of LAG3^+^^38^. LAG3^+^ regulatory T cells are a potent subtype of Tregs known to produce high levels of IL-10^22^ and IL-35^43^. These immunosuppressive mediators were shown to regulate the production of IL-10^+^ as well as IL-35^+^ producing Bregs, which are mostly CD138^+^ cells^10,22,25,44^. In addition, CTLA‐4Ig was also shown to inhibit effector T cells and enhance Tregs activity through a TGF-β dependent mechanism^45^. The above could explain the enhanced differentiation of Tregs and Bregs following abatacept treatment. More work, however, is need to further characterize these mechanisms.

Abatacept expands CD138^+^ CD1d^+^ EBi3^+^ IL-10^+^ Bregs, expressing both IL-35^+^ and IL-10^+^^25,26^. This suggests that these regulatory cells, as well as LAG3^+^ cTregs and LAG3^+^ uTregs, could be major source of the observed elevated levels of serum IL-35 in treated patients. Additionally, this increased levels of IL-35 could also be attributed to the elevated levels of serum IFN-β; as a positive correlation between serum IFN-β and IL-35 levels has been reported in MS patients^46^. The sustained elevation in blood level of these regulatory cells and anti-inflammatory cytokines for 6 months following treatment suggest the effectiveness of abatacept treatment in maintaining a state of disease control in RA patients.

We have shown that abatacept (1) expands IL-35 and IL-10 producing Breg subtype of RA to a level that associates with disease remittance, and (2) expands LAG3^+^ cTreg and LAG3^+^ uTreg of RA at 6 months following treatment. Abatacept has been shown to expand IL-10 producing B cells or IL-10 producing LAG3^+^ Tregs^38^. Our data extend these findings by correlating the effect of abatacept on other subtypes of Breg such as IL-35^+^ Bregs (i35Bregs) production. This result however indicates that more attention should be drawn to the potential indirect regulatory effect of biologics on potent regulatory cells as a way to better control autoimmunity. Moreover, the association of the level of IL-35^+^IL-10^+^ Bregs with the level of patient remittance, in fact, suggest that this subpopulation of regulatory cells may play a key role in controlling inflammation and symptoms of RA patients. The mechanism regulating that, however, requires further investigations.

Nevertheless, due to the sample size limitation and cross-sectional design of our study, this phenomenon and the mechanism regulating it requires further investigations. In this study we only investigated specific subtype of Tregs, while other Treg subtypes such as type 1 T regulatory (Tr1) cells, could also be affected by abatacept treatment and hence may contribute to immune suppression. Furthermore, we have only investigated CD138^+^ subtype of Bregs, while other subtypes which don’t express CD138^+^ and regulate immune response have been identified and may contribute to the observed effect of abatacept treatment^47^. However, the level of Bregs expressing CD138^+^ was significantly higher following abatacept treatment compared to Bregs lacking CD138 expression indicating that abatacept may preferentially expand this subtype of Breg (Supplementary Fig. 7).

In conclusion our study is the first to show that abatacept therapeutic effects extend toward enhancing IL-35 and IL-10 producing Breg subtype which correlates with disease status.

## Supplementary Information

Supplementary Information
