# Author Correction: Abatacept enhances blood regulatory B cells of rheumatoid arthritis patients to a level that associates with disease remittance

**DOI:** 10.1038/s41598-021-87874-9

**Published:** 2021-04-13

**Authors:** Maha Fahad Alenazy, Fatemeh Saheb Sharif-Askari, Mohammed A. Omair, Mohammad S. El-Wetidy, Maha A. Omair, Hussam Mitwalli, Saleh Al-Muhsen, Abeer Al-Masri, Qutayba Hamid, Rabih Halwani

**Affiliations:** 1 Immunology Research Lab, College of Medicine, King Saud University, Riyadh, Saudi Arabia; 2 Department of Physiology, College of Medicine, King Saud University, Riyadh, Saudi Arabia; 3 Sharjah Institute of Medical Research, University of Sharjah, Sharjah, United Arab Emirates; 4 Rheumatology Unit, Department of Medicine, College of Medicine, King Saud University, Riyadh, Saudi Arabia; 5 Department of Statistics and Operations Research, College of Science, King Saud University, Riyadh, Saudi Arabia; 6 Department of Pediatrics, College of Medicine, King Saud University, Riyadh, Saudi Arabia; 7 Department of Clinical Sciences, College of Medicine, University of Sharjah, Sharjah, United Arab Emirates; 8 Meakins-Christie Laboratories, Research Institute of the McGill University Health Center, Montreal, QC Canada; 9 Prince Abdullah Ben Khaled Celiac Disease Chair, College of Medicine, King Saud University, Riyadh, Saudi Arabia

Correction to: *Scientific Reports* https://doi.org/10.1038/s41598-021-83615-0, published online 11 March 2021

The original version of this Article contained an error in the Abstract.

“Abatacept, an inhibitor of CD28 mediated T-cell activation, has been shown to be effective in controlling inflammation during rheumatoid arthritis (RA). However, its effects on immune regulatory B and T cells (Bregs and Tregs) has not been fully explored. Thirty-one RA patients treated with abatacept for ≥ 6 months along with 31 RA patients treated with other modalities as well as 30 healthy controls were recruited. Of these 62 RA patient, 49 (79%) were females with a mean age of 54 ± 12 years and disease duration of 10 ± 6 years. The blood levels of Tregs and Bregs and their production of immunosuppressive cytokines, were determined using FACS analysis and Luminex Multiplex assay. Treatment with abatacept significantly enhanced the blood level of IL-35^+^ IL-10^+^ Bregs (P = 0.0007). Their levels were higher in the blood of remitted patients (DAS28-CRP < 2.6) compared to the unremitted ones (P = 0.0173), 6 months following abatacept treatment initiation. Moreover, abatacept treatment significantly enhanced the blood levels of LAG3^+^ conventional and unconventional Tregs of RA patients. This increase in the blood levels of Bregs and Tregs was accompanied with an elevated serum level of IL-35 and IFN-β in abatacept-treated patients. Therefore, Abatacept efficiency to achieve remittance in RA could be attributed, in part, to its ability to enhance immune regulatory cells, especially IL-135^+^ IL-10^+^ Bregs.”

now reads:

“Abatacept, an inhibitor of CD28 mediated T-cell activation, has been shown to be effective in controlling inflammation during rheumatoid arthritis (RA). However, its effects on immune regulatory B and T cells (Bregs and Tregs) has not been fully explored. Thirty-one RA patients treated with abatacept for ≥ 6 months along with 31 RA patients treated with other modalities as well as 30 healthy controls were recruited. Of these 62 RA patient, 49 (79%) were females with a mean age of 54 ± 12 years and disease duration of 10 ± 6 years. The blood levels of Tregs and Bregs and their production of immunosuppressive cytokines, were determined using FACS analysis and Luminex Multiplex assay. Treatment with abatacept significantly enhanced the blood level of IL-35^+^ IL-10^+^ Bregs (P = 0.0007). Their levels were higher in the blood of remitted patients (DAS28-CRP < 2.6) compared to the unremitted ones (P = 0.0173), 6 months following abatacept treatment initiation. Moreover, abatacept treatment significantly enhanced the blood levels of LAG3^+^ conventional and unconventional Tregs of RA patients. This increase in the blood levels of Bregs and Tregs was accompanied with an elevated serum level of IL-35 and IFN-β in abatacept-treated patients. Therefore, Abatacept efficiency to achieve remittance in RA could be attributed, in part, to its ability to enhance immune regulatory cells, especially IL-35^+^ IL-10^+^ Bregs.”

This error has now been corrected in the PDF and HTML versions of the Article.

