# Supplementary material for: Abatacept enhances blood regulatory B cells of rheumatoid arthritis patients to a level that associates with disease remittance
Source: Sci Rep. 2021 Mar 11;11:5629. doi: 10.1038/s41598-021-83615-0 (PMC7952390; doi:10.1038/s41598-021-83615-0)
Supplement: Supplementary file 1 — Supplementary Information [file 41598_2021_83615_MOESM1_ESM.pdf]

Abatacept Enhances Blood Regulatory B Cells of Rheumatoid Arthritis Patients to a Level that Associates with Disease Remittance

Maha Fahad Alenazy, Fatemeh Saheb Sharif-Askari, Mohammed A. Omair, Mohammed Elwatidy, Maha A. Omair, Hossam Mitwalli, Saleh Al-Muhsen, Abeer Al Masri, Qutayba Hamid, and Rabih Halwani

Supplementary Table 1. Association of blood levels CD138$^+$CD1d$^+$EBi3$^+$IL-10$^+$ Bregs or CD25$^+$Foxp3$^+$LAG3$^+$ Tregs or CD25$^-$Foxp3$^+$LAG3$^+$ uTregs with serum levels of cytokines.

**Cytokines**	**Pearson's correlation coefficient (r)**	***P* value**
**CD138$^+$CD1d$^+$EBi3$^+$IL-10$^+$ Bregs**		
IL-10	0.301	0.317
IL-35	-0.141	0.646
IFN-β	-0.052	0.866
**CD25$^+$Foxp3$^+$LAG3$^+$ Tregs**		
IL-10	0.145	0.637
IL-35	0.771	0.002
IFN-β	0.672	0.012
**CD25$^-$Foxp3$^+$LAG3$^+$ Tregs**		
IL-10	-0.052	0.866
IL-35	0.801	0.001
IFN-β	0.713	0.006

Statistics significant: $P < 0.05$.

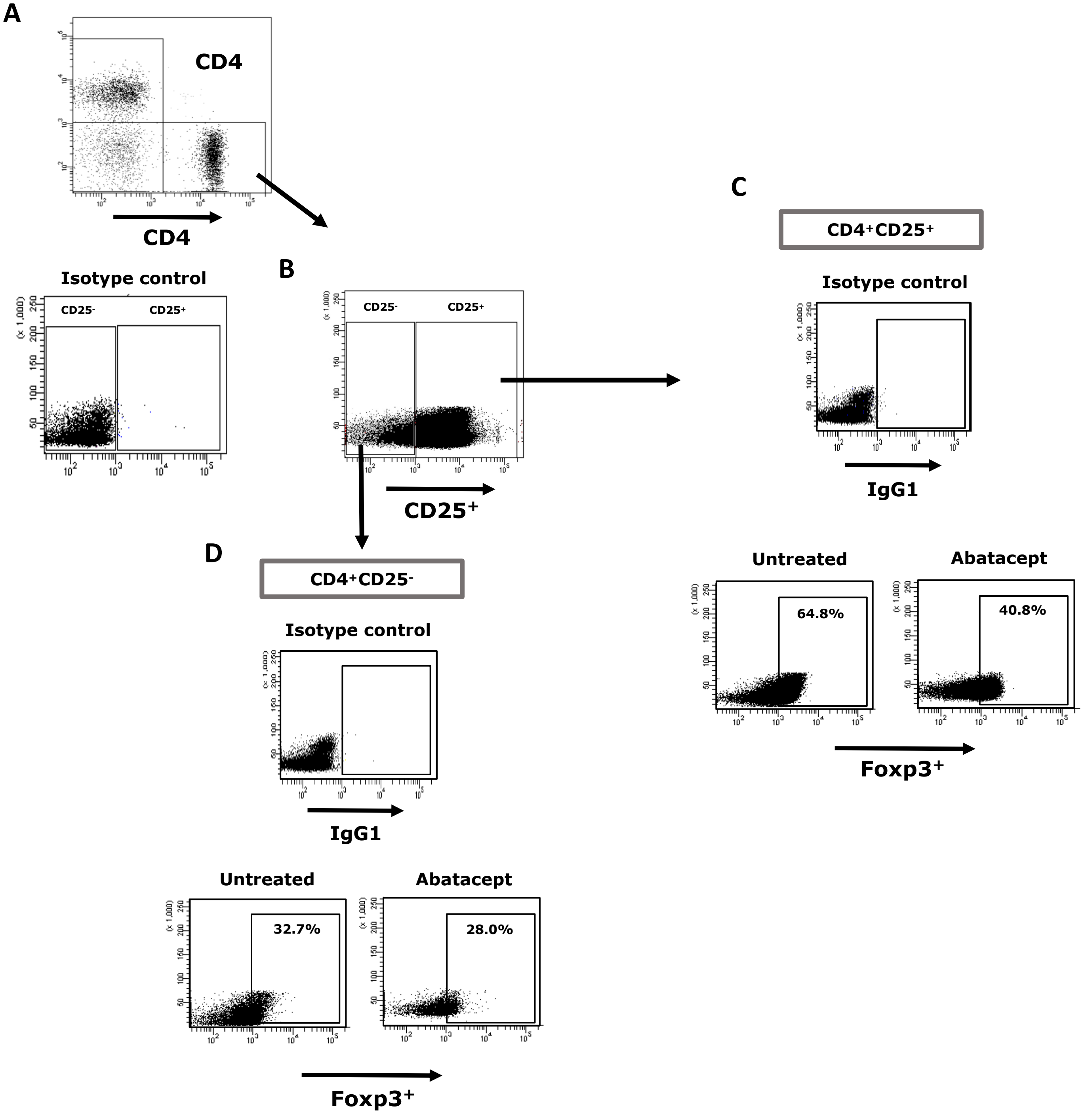

Supplementary Figure 1. Effects of the abatacept on the frequency of conventional Tregs (cTregs) and unconventional Tregs (uTreg) in the PBMCs of RA patients. (A and B) Gating was performed according to CD4 T cells expression and CD25 markers in PBMCs of RA patients. (C) Percentage of cTregs (Foxp3$^+$ cells within CD4$^+$CD25$^+$) in the PBMCs of abatacept-treated and untreated RA patients. Representative FACS data showing no difference in the percentage of these cells in PBMCs following treatment with the abatacept. (D) Percentage of uTregs (Foxp3$^+$ cells within CD4$^+$CD25$^-$) in the PBMCs of abatacept-treated and untreated RA patients. Representative FACS data showing no difference in the percentage of these cells in PBMCs following treatment with the abatacept. Events were recorded and analysed by using BD FACSDiva version 8.0.

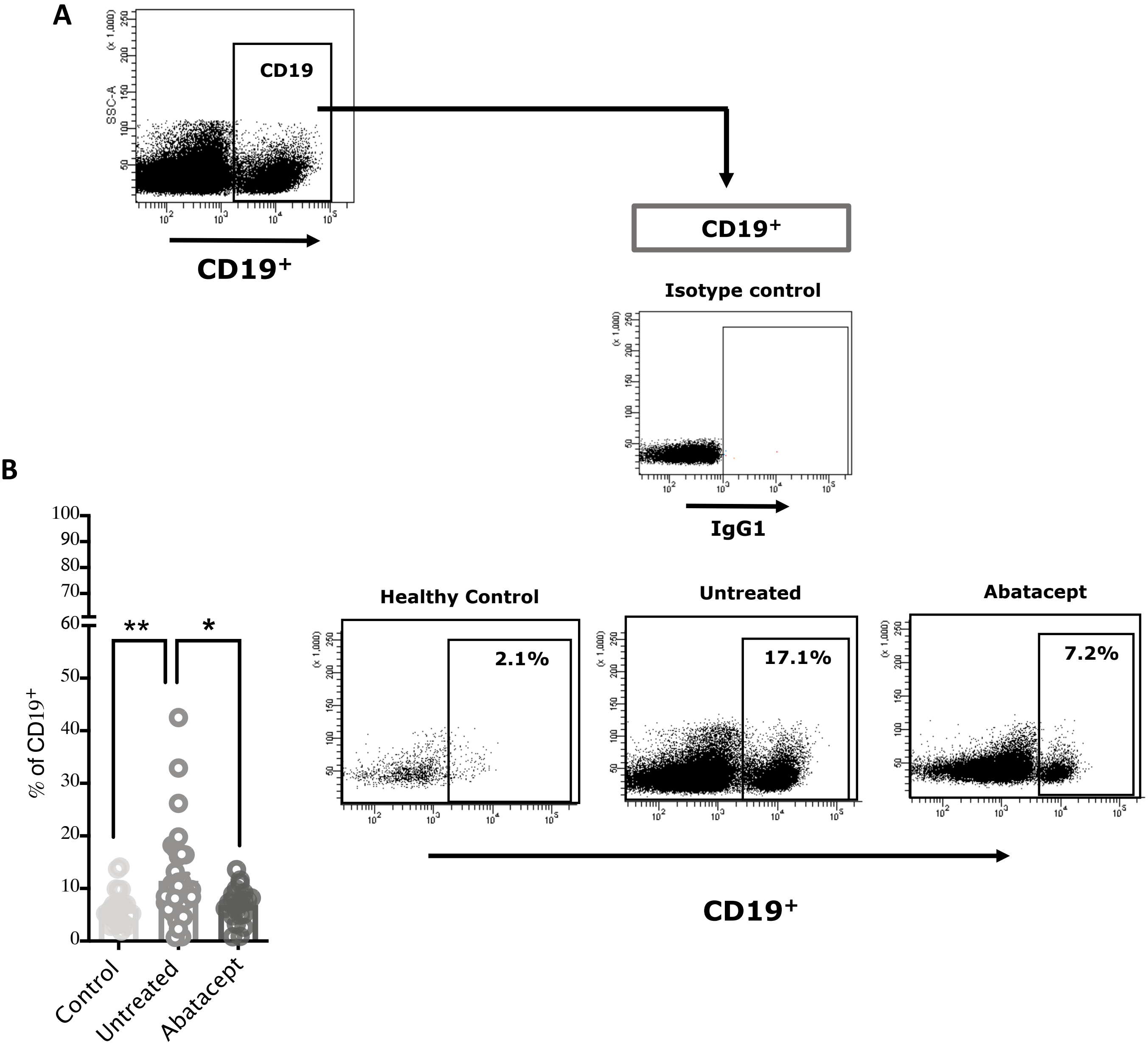

Supplementary Figure 2. Effects of the abatacept on the frequency of $CD19^+$ cells in the PBMCs of RA patients. (A) The gating for $CD19^+$ cells in PBMCs of RA patients. (B) Percentage of $CD19^+$cells in the PBMCs of abatacept-treated and untreated RA patients, and in the healthy controls. Representative FACS data showing a decrease in the percentage of $CD19^+$ in PBMCs following treatment with the abatacept. Events were recorded and analysed by using BD FACSDiva version 8.0. Two-way comparison was done using t-test. * $P<0.05$, ** $P<0.01$.

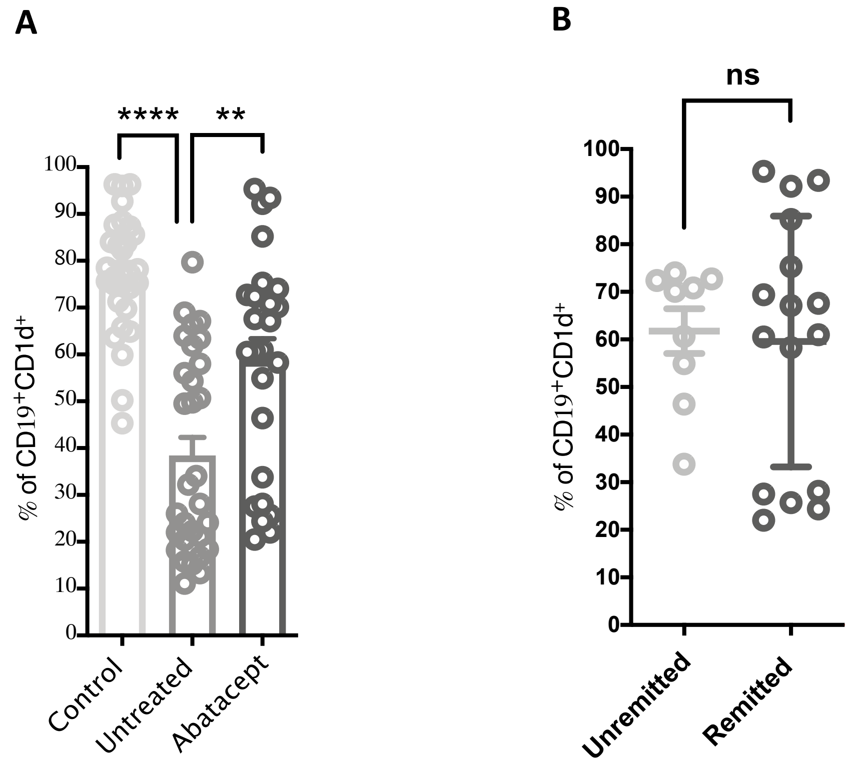

Supplementary Figure 3. Effects of the abatacept on the frequency of CD19^{+}CD1d^{+} Bregs in the PBMCs of RA patients. (A) Percentage of CD19^{+}CD1d^{+} Bregs in the PBMCs of abatacept-treated and untreated RA patients. Representative FACS data showing an increase in the percentage of CD19^{+}CD1d^{+} Bregs in PBMCs following treatment with the abatacept. (B) No different in the blood level of this Breg subtype was observed between remitted and unremitted abatacept-treated groups. DAS28-CRP score lower than 2.6 indicates remission. Two-way comparison was done using t-test. ns: non-significant. ** $P<0.01$, **** $P<0.0001$.

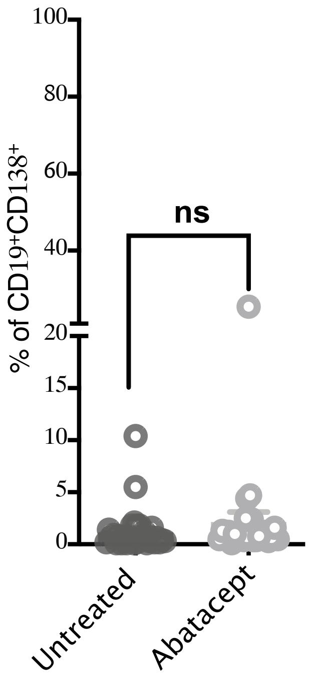

Supplementary Figure 4. Frequency of $CD19^+CD138^+$cells in the PBMCs of abatacept-treated and untreated RA patients. Representative data showing no difference in the percentage of $CD19^+CD138^+$cells in PBMCs following treatment with the abatacept. Two-way comparison was done using t-test. ns: non-significant. * $P<0.05$.

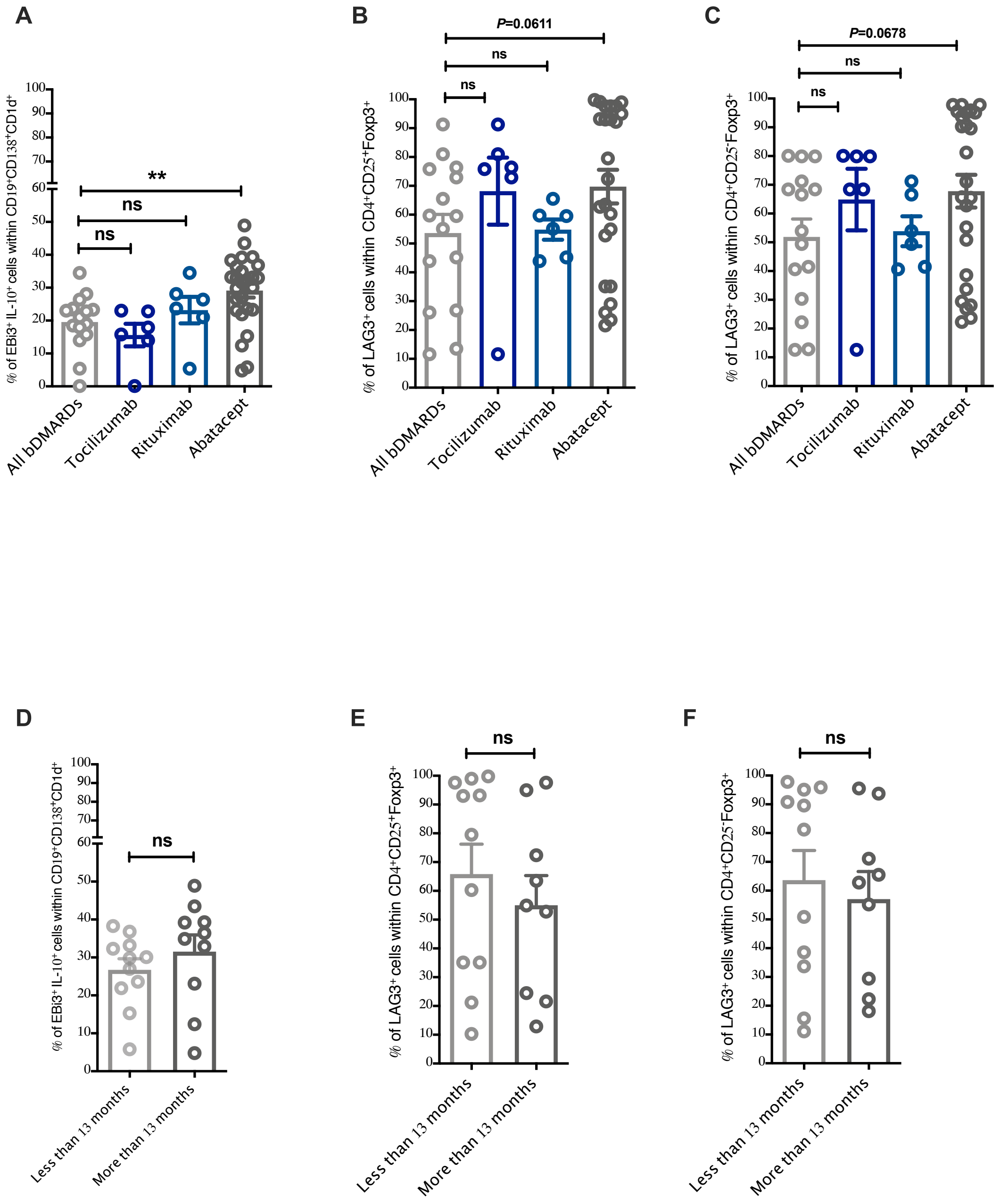

Supplementary Figure 5. Frequency of (A) IL-35$^+$IL-10$^+$ Bregs (B) LAG3$^+$ cTregs, and (C) LAG3$^+$ uTregs in the PBMCs of abatacept-treated patients; or RA patients treated with other bDMARDs, including tocilizumab or rituximab for an average of 13 months. Representative data showing an increase in the percentage of these cells in PBMCs following treatment with the abatacept. Frequency of (D) IL-35$^+$IL-10$^+$ Bregs, (E) LAG3$^+$ cTregs, and (F) LAG3$^+$ uTregs in the PBMCs of abatacept-treated patients who were on more or less than 13 months of therapy. Representative data showing no significant difference in the average frequency of IL-35$^+$IL-10$^+$ Bregs, LAG3$^+$ cTregs, and LAG3$^+$ uTregs between these two groups. Two-way comparison was done using t-test. ns: non-significant. * $P<0.05$, ** $P<0.01$.

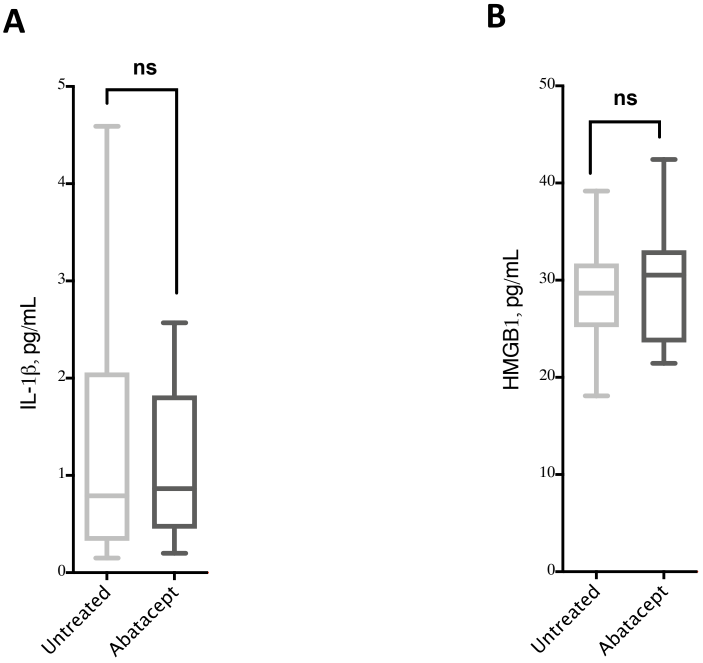

Supplementary Figure 6. Effects of abatacept on the cytokine levels in the serum of RA patients. Serum levels of (A) IL-1β and (B) HMGB1 in abatacept treated and untreated groups. Data show no significant change in of the serum levels of IL-1 β and HMGB1 of abatacept treated compared to untreated RA groups. The cytokine levels were estimated using a human Luminex assay. Two-way comparison was done using t-test. ns: non-significant. * $P<0.05$.

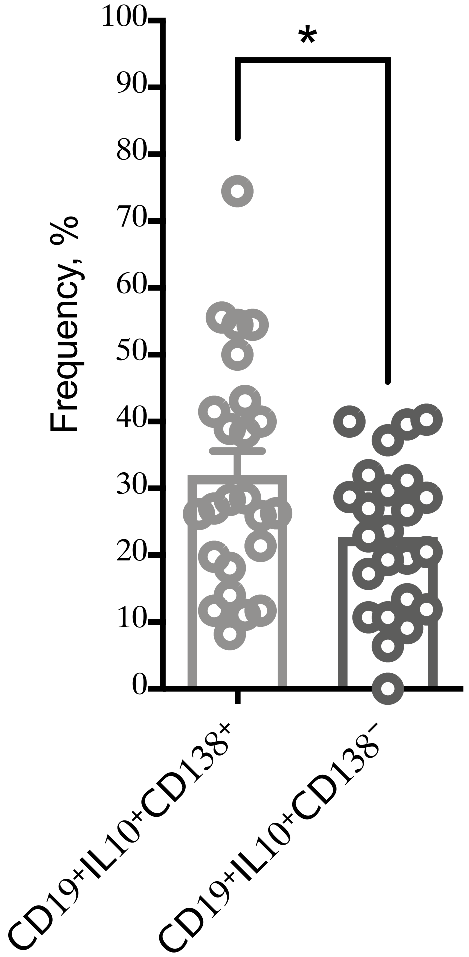

Supplementary Figure 7. Frequency of CD19^{+}IL-10^{+}CD138^{+} and CD19^{+}IL-10^{+}CD138^{-} cells in the PBMCs of abatacept-treated RA patients. Representative data showing an increase in the percentage of CD19^{+}IL-10^{+}CD138^{+} cells in PBMCs following treatment with abatacept. Two-way comparison was done using t-test. * P<0.05.
